# Single-Layer-Graphene-Coated and Gold-Film-Based Surface Plasmon Resonance Prism Coupler Sensor for Immunoglobulin G Detection

**DOI:** 10.3390/s22041362

**Published:** 2022-02-10

**Authors:** Zhe-Wei Yang, Thi-Thu-Hien Pham, Chin-Chi Hsu, Chi-Hsiang Lien, Quoc-Hung Phan

**Affiliations:** 1 Department of Mechanical Engineering, National United University, Miaoli 36063, Taiwan; u0711032@mail.nuu.edu.tw (Z.-W.Y.); cchsu@nuu.edu.tw (C.-C.H.); 2 Department of Biomedical Engineering, International University-Vietnam National University, Ho Chi Minh City 700000, Vietnam; ptthien@hcmiu.edu.vn

**Keywords:** immunoglobulin G detection, surface plasmon resonance, graphene, virus infectious disease

## Abstract

A graphene-based surface plasmon resonance (SPR) prism coupler sensor is proposed for the rapid detection of immunoglobulin G (IgG) antibodies. The feasibility of the proposed sensor is demonstrated by measuring the IgG concentration in phantom mouse and human serum solutions over the range of 0–250 ng/mL. The results show that the circular dichroism and principal fast axis angle of linear birefringence increase in line with increases in IgG concentration over the considered range. Moreover, the proposed device has a resolution of 5–10 ng/mL and a response time of less than three minutes. In general, the sensor provides a promising approach for IgG detection and has significant potential for rapid infectious viral disease testing applications.

## 1. Introduction

Infectious viral diseases such as dengue fever virus, typhoid fever, hepatitis B virus, SARS-CoV-2, or coronavirus disease 2019 (COVID-19) have become a major threat to human life in this century. At present, the major diagnostic method for infectious viral diseases is real-time reverse transcription polymerase chain reaction (RT-PCR). However, the success of RT-PCR is fundamentally dependent on the correctness of the sample collection process. Furthermore, false-negative cases have been consistently reported with RT-PCR testing, which have grave implications for public health by allowing contagious individuals to continue to circulate. Finally, real-time RT-PCR assays are time consuming and need to be performed by well-trained experts in certified laboratories. In theory, the detection of specific antibodies (such as immunoglobulin IgG/IgM) and antigens provides a simple, rapid, reliable, and accessible strategy for the efficient and large-scale screening of suspected cases in point-of-care settings [1,2,3,4]. Many methods have been proposed for virus infectious disease diagnosis based on the detection of IgG/IgM antibodies [5,6,7,8]. Jiang et al. [9] presented the label-free, real-time detection of rabbit IgG antibody over a concentration range of 0–100 μg/mL with a resolution of 0.0625 μg/mL. The authors of [5,6,7,8,9] confirmed the feasibility of infectious viral disease diagnosis based on the detection of IgG/IgM antibodies. However, a fast and reliable IgG/IgM detection method is required. Many methods have been proposed for improving the accuracy and performance of IgG/IgM detection. Graphene-based SPR sensors are among the promising alternatives.

Graphene is a unique material consisting of a planar carbon sheet with a thickness of just one atom [10]. Since its discovery in 2004, many studies have been published on the optical properties of graphene [11,12] and its wide range of applications [13,14], including graphene-based surface plasmon resonance (SPR) sensors with enhanced sensitivity and resolution [15,16]. Graphene is known as the next generation of plasmonic material with a strong field confinement. It is possible to adjust graphene plasmon by modifying its structural and electron properties [17]. Furthermore, the presence of a single graphene layer on gold leads to a strong localization of the field at the graphene–gold interface and drastic field enhancement. Thus, it provides a fourfold enhancement of the electric field in sensing response in conventional SPR [18]. Wu et al. [19] developed a graphene-on-gold SPR sensor incorporating 10 graphene layers, and showed that the sensitivity of the device was around 25% higher than that of a conventional gold-based SPR sensor. Graphene-based SPR sensors have been widely developed for many biosensing applications, including glucose sensing [20] and protein detection [21]. Wu et al. [22] proposed an enhanced graphene-oxide-based SPR sensor for the detection of human IgG antibodies over a range of 0.075–40 μg/mL with a resolution of 5 μg/mL. The authors of [20,21,22] confirmed the feasibility of graphene-based SPR sensors for IgG detection.

Mueller matrix polarimetry is a well established technique for analyzing the anisotropic properties of turbid media [23,24,25]. In 1996, Lu and Chipmen [26] proposed a novel decomposition method to define and compute the diattenuation and retardance of a Mueller matrix for the first time. Since then, the decomposition of Mueller matrix has attracted scientists’ attention and many extended methods have been proposed for solving the polarization calculus [27,28]. In 2013, Guo et al. [29] applied the Mueller matrix polar decomposition method to examine the microscopic structure of samples containing polystyrene microspheres, well-aligned glass fibers, and polyacrylamide in a forward-scattering configuration. However, the Mueller matrix decomposition method proposed in [27,28,29] required a strict sequential order of matrix components. Another alternative technique in which sequential order issuance is overcome, was the differential Mueller matrix method proposed by Azzam in 1978 [30]. However, the differential Mueller matrix is not able to perform reflectance configuration due to the helicity flip of the circular polarization properties [31]. In 2011, Quijano and Diego extended the differential Mueller matrix technique for the reflectance and backscattering measurement of optical properties of turbid media [32,33]. In previous studies [34,35,36], we presented a combination of differential Mueller matrix and surface plasmon resonance (SPR) for extracting the circular birefringence (CB) and circular dichroism (CD) of protein-containing samples.

In the present study, a new single-layer graphene-based SPR sensor was developed for mouse and human IgG detection based on differential Mueller matrix polarimetry. The dual-retarder Mueller polarimetry system from [36] was used for extracting the optical properties of IgG. An SPR prism coupler was employed for creating the total internal reflectance to perform glucose sensing based on polarization absorption. This enabled the utilization of the differential Mueller matrix to perform reflectance configuration. The graphene layer was added to enhance the performance of the sensor based on the outstanding optical properties of graphene. The feasibility of the proposed sensor was evaluated by measuring the CD and principal fast axis angle of linear birefringence (LB) of mouse and human IgG at concentrations in the range of 0–250 ng/mL. The detection time of the proposed technique is approximately 3 min, including data collection.

## 2. Materials

Mouse IgG (SI-I8765), human IgG (SI-I4506), rabbit anti-human IgG (SAB3701275), *N*-(3-dimethylaminopropyl)-*N*-ethylcarbodiimide hydrochloride (03450), *N*-Hydroxysuccinimide (130670), bovine serum albumin (05470), glycine (33226), and phosphate buffered saline (0.01 mol/L, pH = 7.4) were purchased from Sigma Aldrich (St. Louis, MO, USA). d-glucose was purchased from Merck Ltd. (Darmstadt, Germany). All solutions were prepared with deionized water (DI). Poly(methyl methacrylate) (445746) was purchased from Sigma Aldrich (St. Louis, MO, USA), hydrochloric acid (30721) was purchased from Honeywell Fluka (Seelze, Germany), and anisole 99% (A12997) was purchased from Alfa Aesar (Haverhill, MA, USA).

## 3. Graphene-Based SPR Sensor

**Figure 1:**
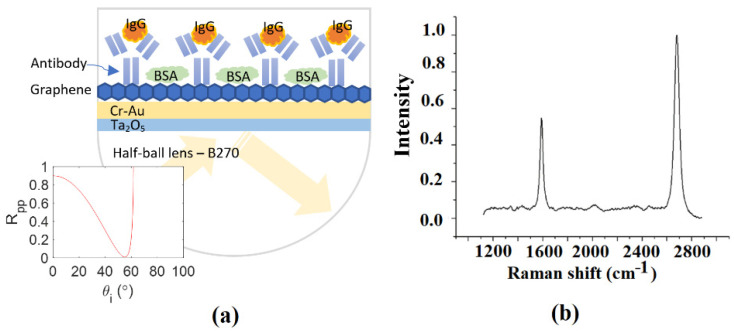
(**a**) Schematic illustration of graphene-based SPR sensor and (**b**) Raman spectroscopy analysis of single-layer graphene.

Figure 1 presents a schematic illustration of the proposed graphene-based SPR sensor. As shown, the sensor comprises a B270 glass half-ball lens (Thorlabs ACL1210U) with a Cr-Au thin film layer (thickness 20 nm) and Ta_2_O_5_ thin film layer (thickness 12 nm) coated on its lower surface, and a single layer of graphene coated on the Cr-Au surface. The glass lens and Cr-Au layer have refractive indices of n_0_ = 1.52 and n_1_ = 0.36 − 2.9i, respectively, while the Ta_2_O_5_ film has refractive indices of n_21_ = 1.637, n_22_ = 1.449, and n_23_ = 1.589 at a wavelength of 633 nm [37]. Moreover, the thickness of the graphene layer is equal to d=L×0.34 nm, where *L* is the number of layers of graphene (one, in the present case); and the refractive index of graphene is $n_{3}=3.0+i(\lambda C/3)$, where *C* is a constant with a value of approximately 5.446 μm^−1^ and *λ* is the wavelength of the incident light [11,12]. It is important to note that one single layer of graphene provides a fourfold enhancement of the electric field in the sensing response [17]. The resonance angle of the SPR sensor was found to be approximately 60° at a wavelength of 632.8 nm. Moreover, the reflectance coefficient, $R_{pp}$, was less than 0.1, as shown in the bottom-left corner of Figure 1a. Note that the reflectance coefficient was calculated using the Berreman 4 × 4 matrix technique for multiple-layer structures [38].

In the graphene-coating process, graphene was grown on a Cu substrate and then transferred to the half-ball lens sensor by placing it in light contact with the surface of the Cr-Au thin film. The graphene was coated on the Cu substrate using the chemical vapor decomposition (CVD) method described in [39]. Briefly, 4 g of PMMA powder was dissolved in 100 mL of anisole (99%, A12997, Alfa Aesar) in a brown jar. The solution was stirred magnetically at 50 °C in a water bath for 6 h, and was then spin-coated onto the Cu substrate at 600 rpm for 6 s and 4000 rpm for 30 s. The Cu substrate was etched in a CuSO_4_: HCl:water solution (10 g:50 mL:50 mL) for 20 min and was then soaked in deionized (DI) water for 10 min and rinsed to wash the deionized water away. The sensor was baked at 100 °C for 20 min, and was then soaked three times in acetone at room temperature to remove the PMMA (note that the soaking times were 3, 5, and 5–10 min, respectively. Finally, the sensor was placed in an oven and dried at 50 °C for 10 min. Figure 1b presents the Raman spectroscopy analysis results for the graphene layer of the SPR sensor. Two prominent peaks were observed at 1600 cm^−1^ and 2700 cm^−1^, respectively, which confirmed that the graphene layer consisted of a single layer of carbon atoms on the Cr-Au surface [40].

For the pretreatment of the graphene-based SPR sensor, a mixed solution of *N*-(3-dimethylaminopropyl)-*N*-ethylcarbodiimide hydrochloride (EDC, 0.4 mol/L, purum grade, ≥98% AT, 03450, Sigma Aldrich) and *N*-Hydroxysuccinimide (NHS, 0.1 mol/L, 98%, 13067, Sigma Aldrich) was filled into a cuvette to completely soak the surface of the sensor for 30 min in order to activate the oxygen-containing functional groups, thereby making it possible to combine it with compounds containing the amino group. The rabbit anti-human IgG solution (250 μg/mL, SAB3701275, Sigma Aldrich) was poured into the cuvette and used to soak the surface of the sensor for 1 h for immobilizing antibody onto the surface of the SPR sensor by covalent and noncovalent bonding. The BSA (10 mg/mL, lyophilized powder, ≥96%, A2153, Sigma Aldrich) was injected into the surface of the sensor for blocking the unbound antibody. Phosphate-buffered saline (PBS, 0.01 mol/L, pH = 7.4, Sigma Aldrich) was injected as the baseline solution. Finally, glycine was used to eluted antibodies and antigens, and the cuvette was washed again by PBS for the next experiment.

## 4. Differential Mueller Matrix Formalism for Extraction of CD/LB Properties of IgG

In the present study, the optical property of the IgG samples was extracted using the Stokes–Mueller polarimetry technique described detail in [36]. The Mueller matrix of the IgG samples was obtained from the Stokes parameters of three input lights with linear polarization states of 0°, 45°, and 90°, respectively, and one input light with right-hand circular polarization. The related equation is given as ${S^{\prime }}_{0{}^{\circ},45{}^{\circ},90{}^{\circ},R}=MS_{0{}^{\circ},45{}^{\circ},90{}^{\circ},R}$, where *S* and *S*′ are the Stokes vectors of the input and output lights, respectively; and *M* is the Mueller matrix. The differential Mueller matrix, *M*, can be expressed using the following eigenvalue analysis formalism [41]:

(1) m=v×(ln(λ)z)×v−1=[m11m12m13m14m21m22m23m24m31m32m33m34m41m42m43m44]

where *ν* and *λ* are the eigenvalues and eigenvectors of the Mueller matrix, respectively, and z is the axis of the coordinate system. For an IgG sample, which has both CD/LB properties and depolarization effects, the differential Mueller matrix can be further expressed as [41]:

(2) $$m=\frac{1}{d}\left[\begin{array}{cccc} \ln\left[\left(1-R^{2}\right)\right] & 0 & 0 & \ln\left(\frac{1+R}{1-R}\right)+\kappa_{v}^{\prime }\\ 0 & \ln\left[\left(1-R^{2}\right)\right]-\kappa_{iq}^{\prime } & 0 & -\beta \sin(2\alpha )+\eta_{u}^{\prime }\\ 0 & 0 & \ln\left[\left(1-R^{2}\right)\right]-\kappa_{iu}^{\prime } & \beta \cos(2\alpha )+\eta_{q}^{\prime }\\ \ln\left(\frac{1+R}{1-R}\right)-\kappa_{v}^{\prime } & \beta \sin(2\alpha )+\eta_{u}^{\prime } & -\beta \cos(2\alpha )+\eta_{q}^{\prime } & \ln\left[\left(1-R^{2}\right)\right]-\kappa_{iv}^{\prime } \end{array}\right]$$

where *d* is the sample thickness, $\kappa_{iq,iu,iv}^{\prime }$ is the diagonal depolarization, $\kappa_{v}^{\prime }$ is the anomalous dichroism, and $\eta_{q,u}^{\prime }$ is the anomalous depolarization. In addition, *R* is the circular dichroism, *α* is the principal fast axis angle of LB, and *β* is the phase retardation of LB. By equating Equations (1) and (2), *R* and *α* can be obtained, respectively, as

(3) $$R=\frac{\exp\left(\frac{m_{14}+m_{41}}{2}\right)-1}{\exp\left(\frac{m_{14}+m_{41}}{2}\right)+1},-1\leq R\leq 1$$

(4) $$\alpha =\frac{1}{2}\tan^{-1}\left(\frac{m_{42}-m_{24}}{m_{34}-m_{43}}\right),0\leq \alpha \leq 180{}^{\circ}$$

## 5. Experimental Setup and Results

**Figure 2:**
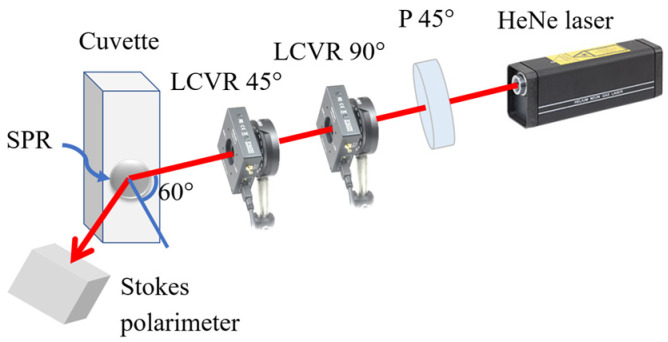
Schematic illustration of dual-retarder Mueller polarimetry system.

In this study, the Stokes–Mueller polarimetry system constructed in the previous study [36] was employed. The illustration schematic of the system is shown in Figure 2. As shown, the main items of equipment included a 633 nm He-Ne laser (1135P, JDS); a polarizer (GTH5M, Thorlabs Inc., Newton, NJ, USA) with the principal axis angle adjusted to 45°; two liquid-crystal variable retarders (LCVRs) (LCC2415VIS/M, Thorlabs Inc., Newton, NJ, USA) with slow axis angles of 90° and 45°, respectively; and a Stokes polarimeter (PAX1000VIS, Thorlabs Inc., Newton, NJ, USA).

The Stokes vector of the light emerging from the polarization state generator (PSG) has the form:

(5) $$S=\mathrm{LCVR}(\delta_{2},45{}^{\circ})\mathrm{LCVR}(\delta_{1},90{}^{\circ}){S^{\prime }}_{in}$$

or,

(6) $$\begin{array}{l} \left[\begin{array}{c} S_{0}^{\prime }\\ S_{1}^{\prime }\\ S_{2}^{\prime }\\ S_{3}^{\prime } \end{array}\right]=\left[\begin{array}{cccc} 1 & 0 & 0 & 0\\ 0 & \cos^{2}(2\theta )+\sin^{2}(2\theta )\cos\delta_{2} & \sin(2\theta )\cos(2\theta )(1-\cos\delta_{2}) & -\sin(2\theta )\sin\delta_{2}\\ 0 & \sin(2\theta )\cos(2\theta )(1-\cos\delta_{2}) & \sin^{2}(2\theta )+\cos^{2}(2\theta )\cos\delta_{2} & \cos(2\theta )\sin\delta_{2}\\ 0 & \sin(2\theta )\sin\delta_{2} & -\cos(2\theta )\sin\delta_{2} & \cos\delta_{2} \end{array}\right]\times \\ \left[\begin{array}{cccc} 1 & 0 & 0 & 0\\ 0 & \cos^{2}(2\theta )+\sin^{2}(2\theta )\cos\delta_{1} & \sin(2\theta )\cos(2\theta )(1-\cos\delta_{1}) & -\sin(2\theta )\sin\delta_{1}\\ 0 & \sin(2\theta )\cos(2\theta )(1-\cos\delta_{1}) & \sin^{2}(2\theta )+\cos^{2}(2\theta )\cos\delta_{1} & \cos(2\theta )\sin\delta_{1}\\ 0 & \sin(2\theta )\sin\delta_{1} & -\cos(2\theta )\sin\delta_{1} & \cos\delta_{1} \end{array}\right]\left[\begin{array}{c} 1\\ 0\\ 1\\ 0 \end{array}\right]\left[\begin{array}{c} S_{0}\\ S_{1}\\ S_{2}\\ S_{3} \end{array}\right] \end{array}$$

where *θ* is the principal axis angle of the LCVRs, and *δ*_1_ and *δ*_2_ are the adjustable phase retardations of the two LCVRs, respectively. Given LCVR principal axis angles of 45° and 90°, Equation (6) can be rewritten as:

(7) $$\left[\begin{array}{c} 1\\ -\sin\delta_{1}\sin\delta_{2}\\ \cos\delta_{1}\\ \sin\delta_{1}\cos\delta_{2} \end{array}\right]=\left[\begin{array}{cccc} 1 & 0 & 0 & 0\\ 0 & \cos\delta_{2} & 0 & -\sin\delta_{2}\\ 0 & 0 & 1 & 0\\ 0 & \sin\delta_{2} & 0 & \cos\delta_{2} \end{array}\right]\left[\begin{array}{cccc} 1 & 0 & 0 & 0\\ 0 & 1 & 0 & 0\\ 0 & 0 & \cos\delta_{1} & -\sin\delta_{1}\\ 0 & 0 & \sin\delta_{1} & \cos\delta_{1} \end{array}\right]\left[\begin{array}{l} 1\\ 0\\ 1\\ 0 \end{array}\right]\left[\begin{array}{l} S_{0}\\ S_{1}\\ S_{2}\\ S_{3} \end{array}\right]$$

The linear polarization lights (0°, 45°, and 90°) and circular polarization light (right hand polarization) required to construct the Mueller matrix of the IgG samples can then be generated by assigning *δ*_1_ and *δ*_2_ the values shown in Table 1. In calibrating the polarimetry system, the principal axis angle of the first LCVR was adjusted to 90° such that the output light was vertical at a phase retardation of 90° and had an orientation angle of 45° at a phase retardation of 0°. The second LCVR was adjusted such that the principal axis angle was orientated at 45°, and the phase retardances of the two LCVRs were then set as shown in Table 1 in order to generate the input lights with the required polarization states. When performing the measurement experiments, the incident angle of the laser light was set equal to the prism coupler resonance angle of 60°. Furthermore, the polarization state of the input light was changed every 2 s. Thus, the average time required to complete the measurement process was approximately 3 min, including data collection. The samples were stored in plastic cuvettes with dimensions of 10 × 10 × 1 mm^3^. The SPR sensor was attached to the cuvettes using industrial glue with a layer of silicon around the base. Prior to mounting the coupler, the cuvettes were drilled with a small hole with a diameter of 6 mm, so that the sample made direct contact with the flat surface of the coupler, thereby avoiding optical interference by the cuvette material.

When performing the experiments, the incident angle of the laser light was set equal to 60°. This was the SPR prism coupler resonance angle. In addition, the sample solution was stored in quartz cuvettes with dimensions of 10 × 10 × 1 mm^3^. Before attaching the coupler to the cuvettes, a small 6 mm—diameter hole was drilled on the cuvettes to ensure that the samples were in direct contact with the half-ball lens flat surface. Thus, the effect of the cuvette material on the measurement results can be ignored.

### 5.1. Determination of IgG Concentration in Mouse Serum Samples

A 1000 ng/mL stock solution of mouse IgG was prepared by adding 2 μL of a buffered aqueous solution of IgG extracted from mouse serum (Sigma Aldrich, SI-I8765, ≥80% SDS-PAGE, concentration: 10~13 mg/dL) to 20 mL of DI water. IgG aqueous solutions with concentrations ranging from 0–250 ng/mL (in 50 ng/mL increments) were prepared by diluting the stock solution with appropriate quantities of DI water (for convenience, the aqueous solutions are referred to as Type 1 samples hereafter). Additional IgG aqueous solutions containing 140 mg/dL D-glucose (100 mg/mL-Merck Ltd.) and 2% lipofundin (lipofundin MCT/LC1 20%, B|Braun, Melsungen, Germany) were additionally prepared to mimic real-world blood plasma with glucose and fatty acid components (for convenience, the samples are referred to as Type 2 samples hereafter).

**Figure 3:**
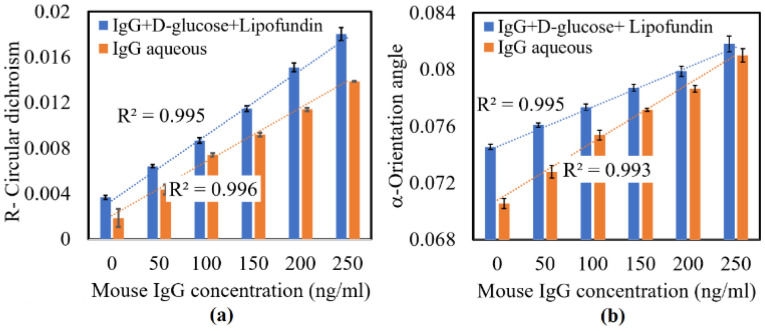
Measurement results obtained for (**a**) circular dichroism (*R*) and (**b**) principal fast axis angle (*α*) of mouse IgG samples with concentrations of 0–250 ng/mL.

Figure 3 shows the experimental results obtained for the circular dichroism (*R*) and principal fast axis angle (*α*) of the various Type 1 and Type 2 samples. As shown in Figure 3a, the value of *R* increased in line with the increasing mouse IgG concentration over the considered range of 0–250 ng/mL for both types of sample. The coefficients of determination were 0.995 (Type 1) and 0.996 (Type 2), respectively. The value of R for the Type 1 samples was smaller than for the Type 2 samples. In other words, the CD property of the Type 2 samples was more strongly corelated with the IgG concentration than that of the Type 1 samples. That is, the addition of glucose and lipofundin to the mouse IgG solution increases the absorption rate of the circular polarization light properties (note that a similar finding was reported in [35], where the CD properties were found to increase in line with the increasing sample complexity). The average standard deviations of the *R* values measured for the Type 1 and Type 2 samples over five repeated tests were found to be 2.9 × 10^−4^ and 4.1 × 10^−4^, respectively. Based on the measured values of *R* for the Type 2 sample, the sensitivity of the measurement results was found to be $S=\Delta R/\Delta C=5.6\times 10^{-5}$ units of R/(ng/mL) where ΔR and ΔC are the variations of the CD and IgG concentration, respectively. The resolution of the measurement results was thus found to be T=δR/S = 5 ng/mL, where δR is the average standard deviation of the measured *R* values.

As shown in Figure 3b, the principal fast axis angle (*α*) of LB also increased in line with the mouse IgG concentration over the considered range of 0–250 ng/mL for both IgG samples with coefficients of determination of 0.995 (Type 1) and 0.993 (Type 2). The measured values of *α* for the Type 1 samples were lower than those of the Type 2 samples. In other words, the addition of glucose and lipofundin increases the principal fast axis angle (*α*) of the sample as a result of the greater linear polarization refraction effect [35]. Moreover, the average standard deviation values of *α* for the Type 1 and Type 2 samples over five repeated tests were found to be 2.8 × 10^−4^ deg and 3.3 × 10^−4^ deg, respectively. Based on the measured values of *α* for the Type 2 samples, the sensitivity of the proposed sensor was found to be S=Δα/ΔC = 3.2 × 10^−5^ units of R/(ng/mL). The resolution of the measurement results was thus obtained as T=δα/S = 10 ng/mL, where δα is the average standard deviation of the measurement results. The resolution of the LB measurement (10 ng/mL) was lower than the resolution obtained for the CD measurement (5 ng/mL). Under total internal reflectance, the graphene exhibited significant absorption in transverse electric mode, which was very sensitive to changes in the refractive index of the media in contact with the graphene. The change in refractive index induced by the interaction of antigens and antibodies resulted in changes in polarization absorption. Thus, the resolution obtained for the CD measurement based on polarization absorption was enhanced by the SPR sensor.

Overall, the results presented in Figure 3 show that the CD and LB properties enable the mouse IgG concentration to be reliably measured with a fine resolution of 5 ng/mL and 10 ng/mL, respectively. Thus, the general feasibility of the proposed sensor for detecting the IgG concentration in mouse serum was confirmed. The applicability of the proposed sensor for IgG detection in human serum was examined by means of further experiments. Note that since the Type 2 sample mimicked the optical properties of real-world blood samples better, the CD and LB measurements were obtained for the Type 2 samples only.

### 5.2. Determination of IgG in Human Serum Ssamples

A 1 mg/mL aqueous solution of human IgG was prepared by dissolving 20 mg of human IgG lyophilized powder (Sigma Aldrich, SI-I4506, ≥95% SDS-PAGE) in 20 mL of DI water. A 1000 ng/mL stock solution of human IgG was prepared by diluting 20 μl of the 1 mg/mL IgG solution with 20 mL of DI water. Human IgG aqueous solutions with concentrations ranging from 0–250 ng/mL (in 50 ng/mL increments), and containing 140 mg/dL D-glucose (100 mg/mL-Merck Ltd.) and 2% lipofundin (lipofundin MCT/LC1 20%, B|Braun), were prepared by diluting the 1000 ng/mL stock solution with appropriate quantities of DI water.

**Figure 4:**
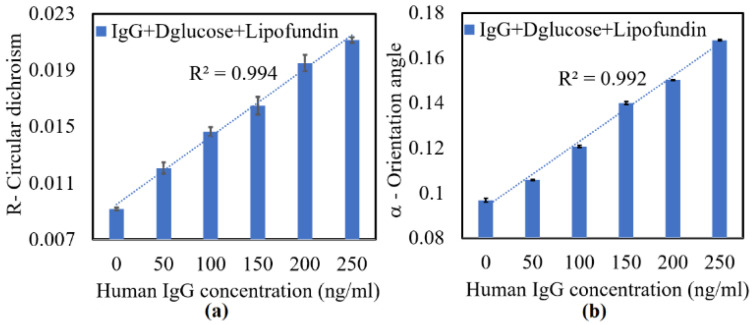
Measurement results obtained for (**a**) circular dichroism (*R*) and (**b**) principal fast axis angle of LB (*α*) of human IgG samples with concentrations of 0–250 ng/mL.

Figure 4 shows the experimental results obtained for the circular dichroism (*R*) and principal fast axis angle (*α*) of the various samples. As shown in Figure 4a, *R* increased linearly with the IgG concentration with a coefficient of determination of 0.994 over the considered range of 0–250 ng/mL. Similarly, as shown in Figure 4b, *α* increased linearly with increasing IgG concentration with a coefficient of determination of 0.992. The average standard deviation values of *R* and *α* over five repeated tests were found to be 3.6 × 10^−4^ and 4.6 × 10^−4^ deg, respectively. Based on the measured values of *R*, the sensitivity of the measurement results was determined to be S=ΔR/ΔC = 6 × 10^−5^ units of *R*/(ng/mL). The resolution of the measurement results was thus found to be T=δR/S = 6.5 ng/mL, where δR is the average standard deviation of the measured CD value. Based on the measured values of *α*, the sensitivity of the measurement results was determined to be S=Δα/ΔC = 4.8 × 10^−5^ unit of *R*/(ng/mL), while the resolution was found to be T=δα/S = 8.1 ng/mL. Thus, as in the mouse IgG samples, the CD measurements provided a better resolution performance than the LB measurements because the polarization absorption were enhanced by the SPR sensor.

Table 2 compares the IgG sensing performance of the proposed graphene-based sensor with that of several other detection methods reported in previous research. As shown, the proposed sensor achieves at least two orders better resolution than the immunoassay magnetic bio separation method proposed in [42], the enzyme-linked immunosorbent assay (ELISA) technique proposed in [43], and the SPR-graphene oxide-based sensors proposed in [7,9]. Moreover, the resolution of the proposed sensor is one order better than that of the ELISA method proposed in [44]. To the best of our knowledge, the sensor reported in the present study is the first sensor to use the differential Mueller matrix formalism to perform a rapid IgG detection.

## 6. Conclusions

This study proposed a novel technique for rapid IgG detection using a single-layer graphene-based SPR sensor. The validity of the proposed sensor was demonstrated by measuring the circular dichroism (*R*) and principal fast axis angle of LB (*α*) of aqueous solutions containing mouse and human IgG antibodies in concentrations of 0–250 ng/mL. The results showed that both properties increased in line with the increasing IgG concentration. Furthermore, for both samples, the magnitude of the CD and LB properties increased with the addition of glucose and lipofundin to mimic the properties of actual human blood. The experimental results showed that the IgG measurement resolution obtained using the CD property is better than that obtained using the LB property because the polarization absorption was enhanced by the SPR sensor. In particular, the CD measurements yielded detection resolutions of 5 ng/mL and 6.5 ng/mL for mouse IgG and human IgG, respectively, whereas the LB measurements yielded equivalent resolutions of 10 ng/mL and 8.1 ng/mL, respectively. It was additionally shown that the standard deviation values of the CD measurements for mouse and human IgG were approximately 4.1 × 10^4^ and 3.6 × 10^−4^, respectively, over five repeated measurements. Similarly, the standard deviation values of the LB measurements for mouse and human IgG were 3.3 × 10^−4^ deg. and 4.6 × 10^−4^ deg, respectively. Overall, the results confirm that the proposed technique provides a promising approach to IgG rapid detection and viral disease diagnosis, including dengue fever virus, Hepatitis B virus, and COVID-19.

## Figures and Tables

**Table 1:** Output polarization states generated by dual-retarder Muller polarimetry system.

Phase Retardation of LCVRs		State of Polarization
δ_1_	δ_2_	
90°	270°	0°
0°	0°	45°
90°	90°	90°
90°	180°	R-

**Table 2:** IgG sensing performance of existing detection methods.

Actual Glucose Concentration (mg/dL)	Sensing Medium	Range of Detection (ng/mL)	Resolution (ng/mL)	Ref.
Immunoassay and magnetic bio-separation	Pig IgG serum	0–25 × 10^3^	5 × 10^3^	[42]
Enzyme-linked immunosorbent assay	Human IgG serum	0–1000	100	[43]
Enzyme-linked immunosorbent assay	Mouse, goat IgG serum	0–100	20	[44]
SPR-graphene oxide	Rabbit IgG serum	0–100 × 10^3^	62.5	[7]
SPR-graphene oxide	Human IgG serum	0–100 × 10^3^	5 × 10^3^	[9]
SPR-single-layer graphene	Human, mouse IgG serum	0–250	5–10	Present study
